# Supplementary material for: An Assessment of Government Capacity Building to Restrict the Marketing of Unhealthy Food and Non-Alcoholic Beverage Products to Children in the Region of the Americas
Source: Int J Environ Res Public Health. 2021 Aug 6;18(16):8324. doi: 10.3390/ijerph18168324 (PMC8394636; doi:10.3390/ijerph18168324)
Supplement: Supplementary file 1 [file ijerph-18-08324-s001.zip › ijerph-1299370 supplementary.pdf]

Supplementary File. 28-item Web-based Survey

Government-capacity to restrict marketing of unhealthy food and non-alcoholic beverages to children in the Region of the Americas

Instructions

Dear Colleague,

Please help to complete the full survey, your input is very valuable. The survey should be completed by a person with knowledge of the food regulatory process, including any regulatory initiatives underway regarding marketing of unhealthy food and beverage products to children, in the country; ideally someone from the food regulatory agency or from the noncommunicable disease program with knowledge of any marketing of unhealthy food and beverage products to children regulatory work. The survey collects information on policies and practices and does not require any field data collection. This questionnaire consists of four sections and three modules with a total of 28 questions. The survey will take approximately 25 minutes to complete, depending on the extent of the regulatory actions on marketing of unhealthy food and beverage products to children in your country.

General Information on those who completed the web-based questionnaire

1. Name and country
2. Position
3. E-mail

Module I. Public Health Infrastructure: This module includes questions related to the presence of a unit or division in the Ministry of Health or other national organization focused on population health, as well as staff and funding. It assesses the existence of a formal multisectoral mechanism to restrict the marketing of unhealthy food and non-alcoholic beverages to children.

4. Is there a unit/branch/department in the Ministry of Health or equivalent with responsibility to restrict the marketing of unhealthy food and beverage products to children in your country? Please specify.

- Yes
- No
- Do not know

Please specify:

5. Indicate the number of full-time equivalent technical/professional staff in the government agency unit, branch or department who are responsible for restricting the marketing of unhealthy food and non-alcoholic beverages to children.

- 0
- 1
- 2-5
- 5 or more
- Do not know

6. Indicate below whether there is funding allocated in the national government's budget to support any policies to restrict the marketing of unhealthy food and non-alcoholic beverage products to children in your country.

	Yes	No	Do not know
• Policy design and implementation			
• Surveillance, monitoring and/or evaluation			
• Capacity-building			
• Research			

7. Indicate the major sources of regular funding for policies, strategies, and actions to restrict the marketing of unhealthy food and non-alcoholic beverages to children in your country.

- General government revenues
- International donors
- Earmarked budgets from taxes on sugar sweetened beverages, alcohol, tobacco, snack foods
- Subsidies for healthy foods such as fruits and vegetables
- Other, please specify below

Please specify:

8. Are there any partnerships or multisectoral collaborations with other institutions, companies, business alliances, industry trade associations, or individuals to enhance the policies, strategies, and actions to restrict the marketing of unhealthy food and non-alcoholic beverages to children in your country?

- Yes
- No
- Do not know

9. Select all the boxes that apply below on partnerships or multisectoral collaborations to restrict the marketing of unhealthy food and non-alcoholic beverages to children in your country.

- Other government Ministries or agencies besides the health sector (i.e., Ministry of Education, Ministry of Finance. Ministry of Social Welfare)
- United Nations agencies (i.e.,The World Bank, Pan-American Health Organization / World Health Organization (WHO), Food and Agricultural Organizations [FAO], United Nations Children's Fund [UNICEF])
- Academia, including any research centers, or universities
- Non-governmental or civil society organizations
- Private-sector firms or organizations (i.e., food and beverage manufacturers, restaurants, retailers, food service companies, entertainment and media companies, corporate foundations, business alliances, and industry trade organizations)
- Do not know

10. Indicate in the box below the type of evidence sources (i.e., reports, studies or other resources) used to develop, implement, or modify policies, strategies, or actions to restrict the marketing of unhealthy food and non-alcoholic beverages to children in your country.

11. Indicate if there are effective leaders, policymakers, champions or advocacy organizations who create strategic directions, motivate staff, and align goals to implement policies, strategies, or actions to restrict the marketing of unhealthy food and non-alcoholic beverages to children in your country. Please specify.

- Yes
- No
- Do not know

Please specify:

Module II. Policy efforts: This module includes questions relating to the presence of policies, strategies, or action plans that restrict the marketing of unhealthy food and non-alcoholic beverages to children in your country.

12. If your country includes policies, strategies or actions to restrict the unhealthy marketing of food and non-alcoholic beverages to children in the national plan to reduce and prevent obesity and/or diet-related non-communicable diseases, please attach file.

Choose File
Web links:

13. Does your country prioritize on the current national agenda any policies, strategies, or actions to restrict the marketing of unhealthy food and non-alcoholic beverages to children?

- Yes
- No
- Do not know

14. List in the box below the specific policies, strategies, or actions to restrict the marketing of unhealthy food and non-alcoholic beverages to children on the current national agenda in your country.

15. Attach files of the policies, strategies, or actions to restrict the marketing of unhealthy food and non-alcoholic beverage products to children on the current national agenda in your country

Choose File
Web links:

16. Does your country have national targets or specific, measurable and time-scaled objectives to restrict the marketing of unhealthy food and non-alcoholic beverages to children under the implementation of the Sustainable Development Goal 3 "Ensure healthy lives and promote wellbeing for all at all ages"?

- Yes
- No
- Do not know

17. Specify the national targets or specific, measurable and time-scaled objectives that restrict the marketing of unhealthy food and non-alcoholic beverages to children in your country.

18. Select all the boxes that apply below on the type of policies, strategies, and actions that restrict the marketing of unhealthy food and non-alcoholic beverages to children in your country.

- Voluntarily/self-regulated by industry
- Government guidelines, legislation or laws
- No policies, strategies or actions
- Do not know

19. Select the boxes that apply below on the marketing strategies and techniques that your country includes to restrict unhealthy food and non-alcoholic beverages to children.

	Mandatory	Voluntarily	None	Do not know
• Branding				
• Cartoon mascots, licensed or media characters, celebrities				
• Direct marketing				
• Point of sale				
• Premium offers				
• Product design and packaging				
• Product placement				
• Sponsorship				

20. Select the boxes that apply below on the communication channels and settings that your country includes in policies, strategies, and actions to restrict the marketing of unhealthy food and non-alcoholic beverages to children.

	Mandatory	Voluntarily	None	Do not know
• Broadcast (TV and radio) and print media				
• Community, sports and special events				
• Digital and social media				
• Food retailers and restaurants				
• Mobile and digital devices				
• Outdoors and transportation				
• Schools				
• Websites				

21. Select the boxes that apply below on the nutrition criteria or guidelines does the policies, strategies, and actions use to restrict the marketing of unhealthy food and non-alcoholic beverages to children in your country.

	Yes	No	Do not know
• National dietary guidelines			
• Independent criteria (i.e., non- commercial or scientific)			
• Nutrient profile model (i.e., PAHO)			
• Food and beverage product categories			
• Company names and brands			

22. Indicate in the box below the explicit language of your national Constitution about your government protecting children's rights to food and health based on the United Nations Convention on the Rights of the Child.

23. Indicate in the box below the body, entity, or institution that is in charge to enforce the policies, strategies, and actions to restrict the marketing of unhealthy food and non-alcoholic beverages to children.

24. Select the boxes below for the accountability mechanisms that your country use to enforce the policies, strategies, and actions to restrict the marketing of unhealthy food and non-alcoholic beverage products to children.

- Fines
- Public complaints
- Verbal warnings
- Media reports
- Do not know
- Other (please specify):

25. Select the boxes that apply below to show the entities that are responsible for overseeing the investigation of complaints and enforcement of policies, strategies, and actions used to restrict the marketing of unhealthy food and non-alcoholic beverages to children in your country.

- Government
- Industry
- Independent regulator
- Other (please specify):

26. Does your country's policies, strategies, and actions to restrict the marketing of unhealthy food and non-alcoholic beverages to children consider the effects of cross-border marketing for other countries or your region? Please specify.

- Yes
- No
- Do not know

Please specify:

Module III: Information systems: The questions in this module assess surveillance related to the policies, actions or strategies to restrict the marketing of unhealthy food and non-alcoholic beverages to children for each country, and whether data are included in their national health reporting systems.

27. Indicate below whether your country has a national surveillance or monitoring system, uses ongoing surveys, or depends on industry or media reports to collect data on the policies, strategies, and actions to restrict the marketing of unhealthy food and non-alcoholic beverages to children?

	Surveillance system	Surveys	Industry and media reports
• Yes			
• No			
• Do not know			

28. Select below all the entities that are responsible to collect the data and report results of the policies, strategies, and actions to restrict the marketing of unhealthy food and non-alcoholic beverages to children in your country.

- Government agencies
- Industry corporations and trade associations
- Independent regulator
- Research institutions
- Public-interest and civil society organizations
- Investigative journalists in the media
- Do not know

This is the end of the survey.
Thank you for your time.
